# Performance Comparison of Al–Ti Master Alloys with Different Microstructures in Grain Refinement of Commercial Purity Aluminum

**DOI:** 10.3390/ma7053663

**Published:** 2014-05-07

**Authors:** Wanwu Ding, Tiandong Xia, Wenjun Zhao

**Affiliations:** 1 School of Materials Science and Engineering, Lanzhou University of Technology, Lanzhou 730050, Gansu, China; E-Mails: dingww@lut.cn (W.D.); zhaowj@lut.cn (W.Z.); 2 State Key Laboratory of Gansu Advanced Non-ferrous Metal Materials, Lanzhou 730050, Gansu, China

**Keywords:** Al–5Ti master alloy, TiAl_3_, grain refining, commercial purity Al

## Abstract

Three types of Al–5Ti master alloys were synthesized by a method of thermal explosion reaction in pure molten aluminum. Performance comparison of Al–5Ti master alloy in grain refinement of commercial purity Al with different additions (0.6%, 1.0%, 1.6%, 2.0%, and 3.0%) and holding time (10, 30, 60 and 120 min) were investigated. The results show that Al–5Ti master alloy with blocky TiAl_3_ particles clearly has better refining efficiency than the master alloy with mixed TiAl_3_ particles and the master alloy with needle-like TiAl_3_ particles. The structures of master alloys, differing by sizes, morphologies and quantities of TiAl_3_ crystals, were found to affect the pattern of the grain refining properties with the holding time. The grain refinement effect was revealed to reduce markedly for master alloys with needle–like TiAl_3_ crystals and to show the further significant improvement at a longer holding time for the master alloy containing both larger needle–like and blocky TiAl_3_ particles. For the master alloy with finer blocky particles, the grain refining effect did not obviously decrease during the whole studied range of the holding time.

## 1. Introduction

The grain refinement of aluminum by addition of Al–Ti and Al–Ti–B master alloys has been widely used and studied in recent years. Much practical and theoretical work has been done to investigate a more effective Al–Ti–B master alloy, while Al–Ti has often been considered as more simple and understandable master alloy [1–3]. In certain areas, Al–Ti master alloys have been used extensively as an alternative to Al–Ti–B grain refiner. TiAl_3_ particle in Al–Ti grain refiner is a more powerful nucleant for α-Al than any other heterogeneous nucleating particle [4–7].

There is little question as to the mechanism of grain refinement in aluminum by Al–Ti, which is explained by TiAl3 acting as heterogeneous nucleating centers [1] as well as by peritectic theory. The size, morphology and quantity of the nuclei seem to be an important factor in determining the grain refining response of master alloys [8]. Most of the works on grain refining performance of Al–Ti and Al–Ti–B master alloys have been devoted to the influence of their chemical composition, parent–metal composition and processing parameters such as holding temperature, contact time, mechanical agitation and cooling rate on a grain refiner effectiveness [1,3,9]. However, some researchers have considered the effect of microstructure of master alloys on their grain refining response [2,10] and even used special techniques to reduce intermetallic crystals in master alloys in size for the improvement of their efficacy [4,11,12]. The purpose of this investigation was to examine the effect of various microstructures on grain refining performance of Al–Ti master alloys produced by thermal explosion reaction in pure molten aluminum at different temperature.

## 2. Results and Discussion

### 2.1. Preparation of Al–5Ti Master Alloy

**Figure 1.**
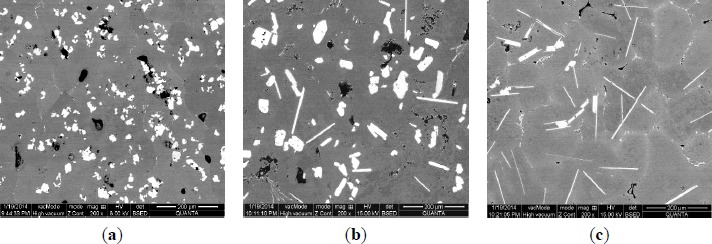
Microstructures of three types of Al–5Ti master alloy: (**a**) master alloy 1^#^ with blocky TiAl_3_ particles; (**b**) master alloy 2^#^ with mixed TiAl_3_ particles; (**c**) master alloy 3^#^ with needle–like TiAl_3_ particles.

**Figure 2.**
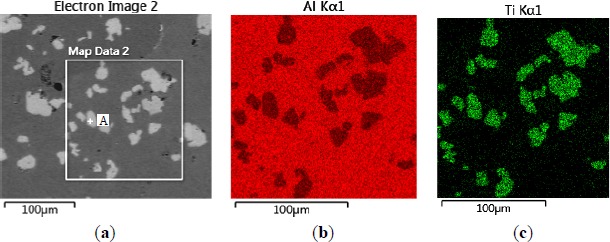

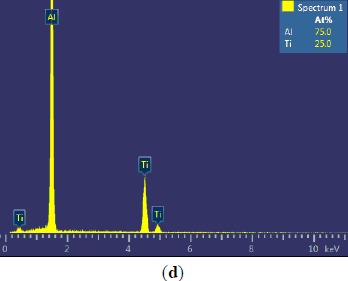
High power SEM image of Figure 1a and elemental maps and EDS analysis of master alloy 1^#^ with blocky TiAl_3_ particles: (**a**) SEM image; (**b**) Al element; (**c**) Ti element; (**d**) EDS analysis of point A in Figure 2a.

**Figure 3.**
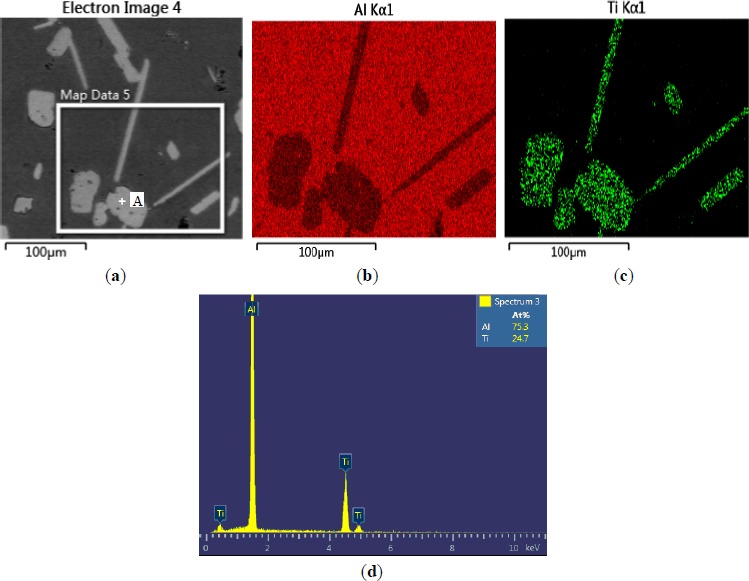
High power SEM image of Figure 1b and elemental maps and EDS analysis of master alloy 2^#^ with mixed TiAl_3_ particles: (**a**) SEM image; (**b**) Al element; (**c**) Ti element; (**d**) EDS analysis of point A in Figure 3a.

**Figure 4.**
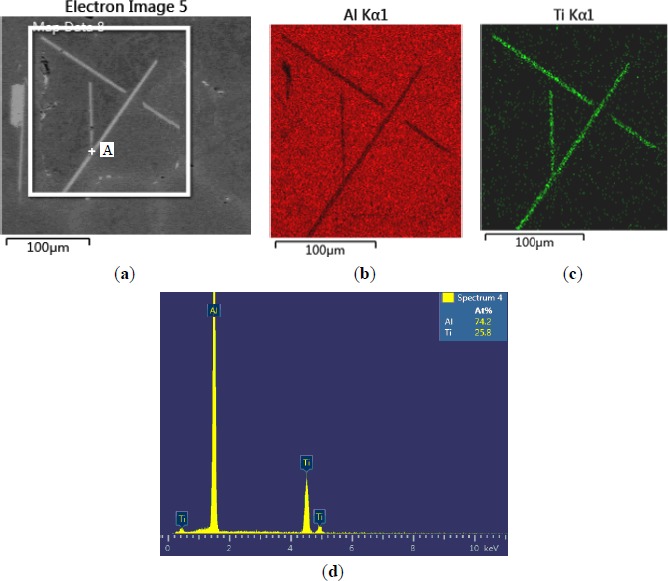
High power SEM image of Figure 1c and elemental maps and EDS analysis of master alloy 3^#^ with needle–like TiAl_3_ particles: (**a**) SEM image; (**b**) Al element; (**c**) Ti element; (**d**) EDS analysis of point A in Figure 4a.

The microstructures of three types of Al–5Ti master alloys synthesized in the experiment are shown in Figures 1–4. Figure 1a shows the SEM image of Al–5Ti master alloy 1^#^. As can be seen, on the Al substrate of Al–5Ti master alloy 1^#^ a large number of blocky particles is uniformly distributed with the average length being roughly 25 μm (Table 1). Figure 2 is high power SEM image of Figure 1a and elemental maps and EDS (Energy Dispersive Spectrometer) analysis of master alloy 1^#^ with blocky particles. Figure 2b,c indicate that blocky particles are rich in element Ti and poor in Al. From Figure 2d, we can see that at point A in the blocky particles, the molar mass fraction of element Al is 75.0%, the molar mass fraction of element Ti is 25.0%, and the molar mass ratio between element Al and element Ti is 3. According to the analysis results of phase composition (Table 2), we can be sure that the blocky particles in Figure 1a are TiAl_3_. Using the same analysis method, we can be sure that the Al–5Ti master alloy 2^#^ has both needle-like and blocky appearances (here called mixed morphology) TiAl_3_ particles with the average length is roughly 40 μm, and Al–5Ti master alloy 3^#^ is composed of needle–like TiAl_3_ particles with an average length of roughly 75 μm.

**Figure 5.**
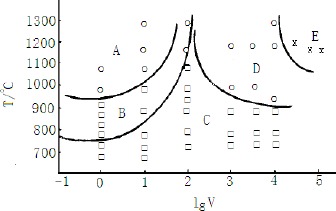
Relationship of the TiAl_3_ morphology with the melting temperature and cooling rate [14]. (**A**) needle–like TiAl3 area; (**B**) mixture of blocky and needle–like TiAl_3_ area; (**C**) blocky TiAl_3_ area; (**D**) metastable area; (**E**) Ti supersatural solution area.

Arnberg [13] and Liu Xiangfa *et al.* [14] think that the morphologies of TiAl_3_ particles is related to the temperature of molten aluminum. Blocky TiAl_3_ could be obtained at low temperature (<850 °C), and needle plate/strip TiAl_3_ could be easily obtained at high melting temperature (higher than 1000 °C). Combined with the cooling rate, the TiAl_3_ form is divided into five regions, as shown in Figure 5 [14]. The block TiAl_3_ phase showed more fusible solution, diffusion, and needle like TiAl_3_ effect of refining time delay. In this paper, the experimental results are consistent with those reported in the literature. TiAl_3_ crystals are a tetragonal crystal system [15]. Blocky TiAl_3_ crystals were formed in the condition of Ti supersaturated crystallization. The TiAl_3_ nucleation driving force was enough to form atoms in the crystal and original surface nucleation because of Ti supersaturated state, which makes the fundamental equilibrium of each crystal TiAl_3_ increase and causes massive growth in three–dimensions. Therefore, in this experiment, master alloy 1^#^ prepared at 800 degrees contains a large number of blocky particles. The blocky and needle-like TiAl_3_ in Master alloy 2^#^ and 3^#^ prepared at the higher temperature, dissolved gradually and resulted in Ti in a supersaturated state. From the thermodynamic stability of the particle, the TiAl_3_ crystallization again formed a larger sized TiAl_3_. Needle flake TiAl_3_ are two-dimensional dendrite crystals, marked the 100, 010, 110 crystal orientation, such that the direction of the growth rate is the same. However, due to the atomic density maximum 001 crystal, the direction of 001 growth rate is lowest [15]; therefore, needle flake TiAl_3_ was formed. In the process of heat preservation, the larger needle flake TiAl_3_ formed smaller TiAl_3_ particles by dissolving and recrystallization.

### 2.2. Refining Performance of Al–5Ti Master Alloy

**Figure 6.**
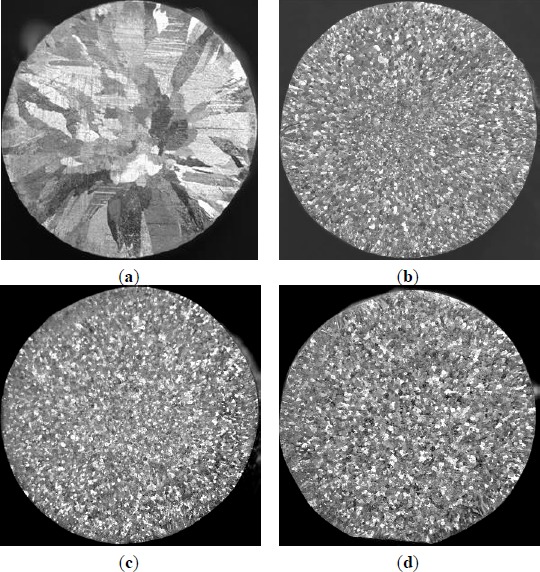
Macrostructures of commercial pure aluminum refined with three types of Al–5Ti master alloys (holding for 5 min): (**a**) Commercial pure aluminum; (**b**) 0.6% master alloy 1^#^; (**c**) 0.6% master alloy 2^#^; and (**d**) 0.6% master alloy 3^#^.

The performance comparison of these master alloys was made in commercial pure aluminum. The grain size comparison of commercial pure aluminum without refiner and including the same content of Al–5Ti master alloys is shown in Figure 6. The macrostructures of commercial pure aluminum samples refined with three types of Al–5Ti master alloys (holding for 5 min) respectively confirm the coarse columnar to equiaxed transition that occurred immediately after the addition of 0.6% Al–5Ti master alloy. Arnberg and Backerud [16] found there are a total of 11 coherent crystal with degree of mismatch no more than 5% between TiAl_3_ particles and α–Al, such as (100)TiAl_3_//(100)Al. Mohanty *et al*. [17] and Cartney [18] found TiAl_3_ particles in the center of the α–Al grain. This shows TiAl_3_ particles could have an aluminum heterogeneous nucleation core. When Al–5Ti master alloys were added to the commercial pure Al melt, a large number of TiAl_3_ particles can be used as nucleation core. The initial quantities of TiAl_3_ also have an effect on the grain refinement result. In the same volume of molten aluminum, due to the increased number of crystal nucleus and make the aluminum grain refinement. The quantity of master alloy 2^#^ is more than two times less than that in the master alloy 1^#^. As seen in Figure 6b–d, Al–5Ti master alloy 1^#^ clearly froms a better refining efficiency than the other two master alloys.

**Figure 7.**
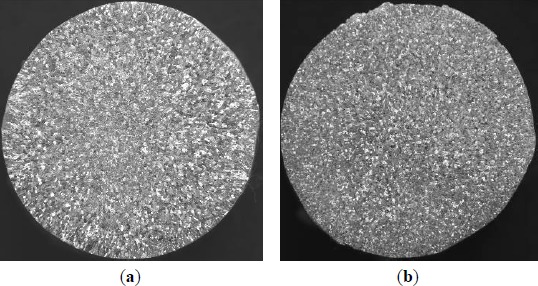

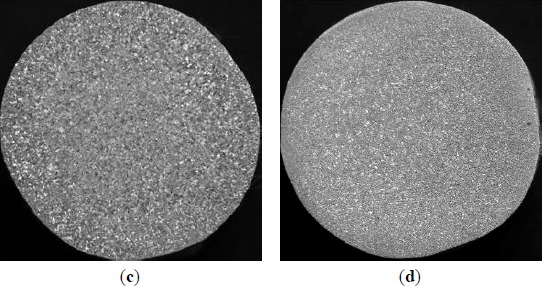
Effects of different additions of Al–5Ti master alloy 1^#^ on grain size of solidified commercial pure aluminum sample (holding for 5 min): (**a**) 1.0%; (**b**) 1.6%; (**c**) 2.0%; (**d**) 3.0%.

**Figure 8.**
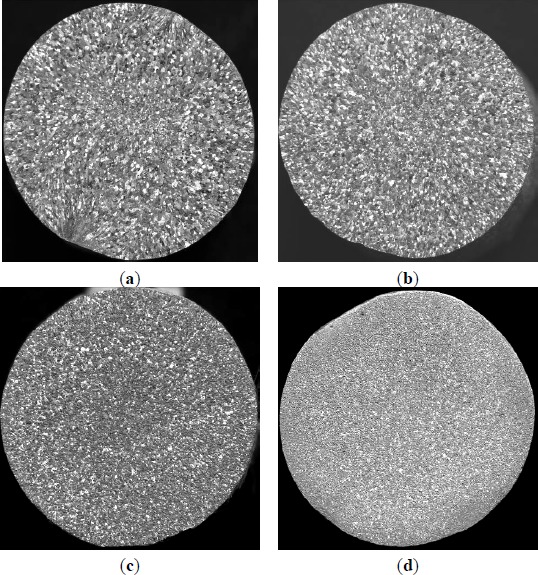
Effects of different additions of Al–5Ti master alloy 2^#^ on grain size of solidified commercial pure aluminum sample (holding for 5 min): (**a**) 1.0%; (**b**) 1.6%; (**c**) 2.0%; (**d**) 3.0%.

**Figure 9.**
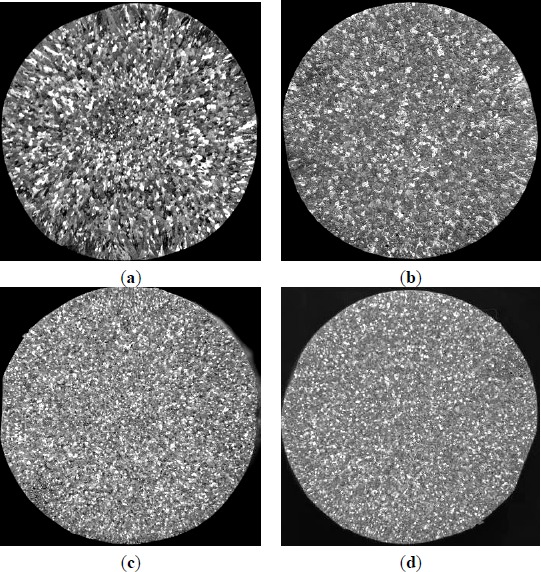
Effects of different additions of Al–5Ti master alloy 3^#^ on grain size of solidified commercial pure aluminum sample (holding for 5 min): (**a**) 1.0%; (**b**) 1.6%; (**c**) 2.0%; (**d**) 3.0%.

**Figure 10.**
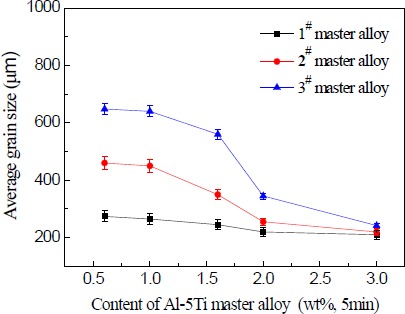
The relation curve between the content of Al–5Ti master alloy and the average size of solidified commercial pure aluminum samples.

In continued studies, the effects of different additions of Al–5Ti master alloy on grain size of solidified commercial pure aluminum sample were detected (Figures 7–9). Even at lower addition level of 1.0%, the master alloy 1^#^ shows good grain refinement as shown in Figure 7a. With increasing addition level to 1.6%, 2.0% and 3.0%, there is an obvious change on the equiaxed grain size (Figure 7b–d). When the addition of Al–5Ti master alloy 2^#^ and 3^#^ was further increased from 1.0% to 3.0%, further improvement in grain refinement is visible from Figure 8a–d and Figure 9a–d respectively. However, as seen in Figures 7–9, Al–5Ti master alloy 1^#^ clearly has better refining efficiency than the other two master alloys when including same content of Al–5Ti master alloy. To illustrate the better performance of three types of Al–5Ti master alloys, Figure 10 shows the quantitative average grain size analysis. It could be clearly seen that, when the addition level of Al–5Ti master alloy 1^#^, 2^#^ and 3^#^ is 1.6%, 2.0% and 3.0% respectively, they showed slightly similar grain refining behavior and the average grain size is roughly 248 μm.

The drop in average grain size at 2.0 wt% master alloy content (Figure 10) could be explained as follows. When Al–5Ti master alloy is added into aluminum melt, aluminum substrate dissolves, and TiAl_3_ particles exist independently in a fused mass. Because, in case of a lower concentration of Ti, TiAl_3_ particles will be unstable, and they will be decomposed into Ti atoms and Al atoms in the melt. When there is a higher concentration of Ti, TiAl_3_ particles will exist stably for a long time in the melt [19–21]. Therefore, before the addition, the amount of grain refining agent of Al–5Ti master alloy reaches 2.0 wt%, most of the TiAl_3_ particles added will dissolve into the melt, so they can not exist stably and become cores of heterogeneous nucleation, thus having a slightly better refining effect. When Al–5Ti master alloy reaches 2.0 wt%, with the increase in the addition amount of refinement agent, the TiAl_3_ particles added can exist stably for a long time in the melt and become cores of heterogeneous nucleation during crystallization of α-Al. Thus, the sample grains are refined significantly until after the addition amount of refining agent reaches 3.0 wt% master alloy content. At this point, there exist in the melt a sufficient number of TiAl_3_ particles and the refining effect reaches a saturated state.

**Figure 11.**
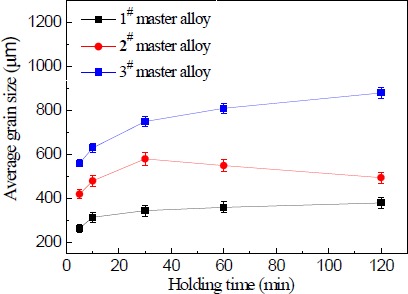
The relation curve between holding time and the average size of solidified commercial pure aluminum samples refined with 1.0 wt% master alloy 1^#^–3^#^.

Figure 11 shows the relation curve between holding time and the average size of solidified commercial pure aluminum samples refined with 1.0 wt% master alloy 1^#^–3^#^. It is clear that the different master alloys were found to show large differences in the grain refining properties with the holding time. As seen in Figure 11, the grain refinement deteriorated as the holding time increased when the master alloy 3^#^ contained needle–like TiAl_3_ crystals. In the worst case, at 120 min, the average grain size increased by a factor of about two in comparison with 5 min level. The master alloy 2^#^ containing both needle–like and blocky TiAl_3_ particles also resulted in a deterioration of the grain refining properties and the holding time increased from 5 to 30 min. At 30 min, the average grain size increased by a factor of one point four, and at 120 min, it sharply reduced to the 10 min level.

**Figure 12.**
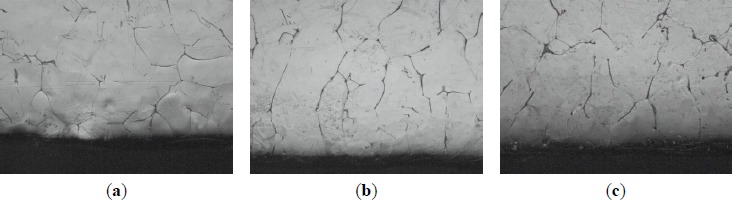
Macrostructures at the bottom of commercial pure aluminum refined with three types of Al–5Ti master alloys (holding for 120 min): (**a**) 1.0 wt% master alloy 1^#^; (**b**) 1.0 wt% master alloy 2^#^; and (**c**) 1.0 wt% master alloy 3^#^.

Figure 12 shows macrostructures at the bottom of commercial pure aluminum refined with three types of Al–5Ti master alloys holding for 120 min. It can be seen from Figure 12a–c that after a heat treatment time of 120 min, small lump-like or strip-like TiAl_3_ is not found in the precipitates of the refinement sample, which is due to the fact that in the case of less addition amount of alloy, the TiAl_3_ added into the aluminum melt has been melted and dissolved into the melt before it precipitates to the bottom of the sample and becomes the solute Ti [21] in the aluminum melt. The grain size reduction after 30 min of the holding time for master alloy 2^#^ could be explained as follows. Staying longer in the melt, TiAl_3_ crystals gradually start to dissolve that leads to the reductions in their size and to the fragmentation of larger particles into smaller ones, which caused increase the number of TiAl_3_ particles. Because the number of TiAl_3_ particles is increased, the aluminum melt nuclei also increased. In the solidification process of aluminum melt, small particles of TiAl_3_ crystals could be the heterogeneous nuclei. Therefore, solidification sample of smaller grain size was obtained. Master alloy 1^#^ contains a large number of blocky particles that impedes their fast dissolution and does not provide a obvious change on the equiaxed grain size at least within the studied range of the holding time. The initial quantities of TiAl_3_ also have an effect on the grain refinement result—their quantity in master alloy 2^#^ is more than two times less than that in master alloy 1^#^.

These results clearly show that TiAl_3_ particle size plays an important role on their dissolution tendency in molten Al. TiAl_3_ particle is known to be a powerful nucleant for α-A1 [4]. However, when added in the hypoperitectic composition during grain refinement, they dissolve in molten Al and cause significant fading. The literature has shown that the time required for complete dissolution depends on dissolution kinetics, which depends on the diffusivity of Ti into Al, particle size and temperature [4]. In our present study we showed the dissolution behavior of TiAl_3_ particles of different size and distribution in molten Al held at constant temperature and different holding time. The results obtained are summarized as follows: (1) Significant fading is observed on longer holding with needle-like TiAl_3_ particles; (2) Master alloy 2^#^ with mixed TiAl_3_ particles has relatively wider particles size distribution in comparison to other two master alloys, and TiAl_3_ particles are not completely dissolved within the holding time 30 min; (3) For master alloy 1^#^ with blocky TiAl_3_ particles, fading is minimum, suggesting much less particle dissolution in comparison to other Al–5Ti master alloys.

## 3. Experimental Section

The three types of Al–5Ti master alloys were synthesized by a method of thermal explosion reaction in the pure molten aluminum. The Al–5Ti master alloys were produced by reacting prefabricated blocks with molten aluminum. The prefabricated blocks were made through ball mixing and cold pressing of the main raw materials of Al powder (99.6%) and Ti powder (99.3%). The supplier and particle size of the powders have been given in Table 3. Commercial purity aluminum ingot was first cut and heated to a required temperature in a graphite crucible using a resistance furnace to hold the melt isothermally at 800, 1000 and 1000 °C respectively. It was then weighed to achieve a composition of Al–5Ti in the alloy. It was compacted to a cylindrical prefabricated block and added into the aluminum melt and left for 10, 10 and 30 min without introducing any stirring. Subsequently, the melts were stirred with a graphite rod for 30 s and mixed thoroughly. The mixed alloys were finally cast into a steel mold. Samples were sectioned from the final alloys and then prepared with standard metallographic procedure. The master alloys were characterized by RigakuD/max–A X–ray diffract meter (XRD, PW 3040/60, PANalytical, Rotterdam, The Netherlands), JSM–7500 scanning electron microscope (SEM, SSX–550 fitted with EDS equipment, Shimadzu Corporation, Kyoto, Japan) after etching with Busswell’s reagent. The synthesis parameters and composition of Al–5Ti master alloys have been given in Table 2.

For grain refinement, 150 g of commercial pure Al (99.97% purity, The Northwest Aluminum Company, Dingxi, Gansu, China) was melted in an electrical resistance furnace. The melt was brought to a temperature of 720 °C. Subsequently, the different additions of Al–5Ti master alloy (0.6%, 1.0%, 1.6%, 2.0%, and 3.0%) were added to the commercial pure Al melt, stirred thoroughly and held temperature for 5 min to ensure homogeneity of composition. In order to study the holding time on the grain refining effciency of commercial pure Al, 1.0 wt% Al–5Ti master alloy was added to the commercial pure Al melt, stirred thoroughly and held for 10, 30, 60 and 120 min at 720 °C respectively. After the slag of the melt was skimmed, the melt was poured into a cylindrical steel mold with size of 50 mm × 25 mm on a fire clay brick. In order to investigate the precipitates to the bottom of the sample, the refined samples, which were made through the above method and preserved for 120 min were naturally cooled in the crucible. All of the samples were etched by a reagent (60% HCl + 30% HNO_3_ + 5% HF + 5% H_2_O, the compositions here are in volume fraction). Lastly, images were taken for each sample to analyze their macrostructures. The grain sizes were measured with the linear intercept method.

## 4. Conclusions

Three types of Al–5Ti master alloys were synthesized and grain refining performance of Al–5Ti master alloy with different additions and holding time were studied. The following conclusions were drawn:

(i) Three types of Al–5Ti master alloys with blocky TiAl_3_ particles, mixed TiAl_3_ particles and needle-like TiAl_3_ particles were successfully prepared through a method of thermal explosion reaction in the pure molten aluminum.
(ii) Al–5Ti master alloy with blocky TiAl_3_ particles clearly has better refining efficiency than the master alloy with mixed TiAl_3_ particles and with needle-like TiAl_3_ particles when including same content of Al–5Ti master alloy.
(iii) The sizes, morphologies and quantities of TiAl_3_ crystals of Al–5Ti master alloys were found to affect the pattern of the grain refining properties with the holding time. The grain refinement effect was revealed to decrease markedly for needle-like TiAl_3_ crystals and to show the further significant improvement at a longer holding time for both larger needle-like and blocky TiAl_3_ particles. For the finer blocky particles, the grain refining effect did not show an obvious decrease during the whole studied range of time.

## Figures and Tables

**Table 1.** Microstructural parameters of TiAl_3_ particles in Al–5Ti master alloys.

Alloy	Morophology	Average length, μm	Include density, cm^−3^
1^#^ master alloy	Blocky	25	47,500
2^#^ master alloy	Mixed	40	22,500
3^#^ master alloy	Needle–like	75	12,500

**Table 2.** The synthesis parameters and composition of Al–5Ti master alloys.

No.	Alloy	Reaction Temperature (°C)	Reaction Time (min)	Phase composition	Nominal Ti (%)
1^#^	Al–5Ti	800	10	TiAl_3_	5
2^#^	Al–5Ti	1000	10	TiAl_3_	5
3^#^	Al–5Ti	1000	30	TiAl_3_	5

**Table 3.** Characteristics of powders.

Materials	Supplier	Particle size/μm	Purity/%
Al powder	The Northwest Aluminum Company	61–74	99.6
Ti powder	Shangxi Baoji state Construction Pioneer Metals Corporation	38–44	99.3
